# Applications of Nanomaterials for Immunosensing

**DOI:** 10.3390/bios8040104

**Published:** 2018-11-01

**Authors:** Sandra Lara, André Perez-Potti

**Affiliations:** Centre for BioNano Interactions, School of Chemistry, University College Dublin, D04 V1W8 Dublin, Ireland

**Keywords:** nanomaterials, immunosensors, biomarkers, antigen, antibody, immune complex, cancer, therapeutics, diagnostics

## Abstract

In biomedical science among several other growing fields, the detection of specific biological agents or biomolecular markers, from biological samples is crucial for early diagnosis and decision-making in terms of appropriate treatment, influencing survival rates. In this regard, immunosensors are based on specific antibody-antigen interactions, forming a stable immune complex. The antigen-specific detection antibodies (i.e., biomolecular recognition element) are generally immobilized on the nanomaterial surfaces and their interaction with the biomolecular markers or antigens produces a physico-chemical response that modulates the signal readout. Lowering the detection limits for particular biomolecules is one of the key parameters when designing immunosensors. Thus, their design by combining the specificity and versatility of antibodies with the intrinsic properties of nanomaterials offers a plethora of opportunities for clinical diagnosis. In this review, we show a comprehensive set of recent developments in the field of nanoimmunosensors and how they are progressing the detection and validation for a wide range of different biomarkers in multiple diseases and what are some drawbacks and considerations of the uses of such devices and their expansion.

## 1. Introduction

Nanomaterials are objects in the size range of 1 nm to 100 nm [1] and as a result of their small dimensions at the nanoscale, they present physico-chemical properties and functions which differ from those seen in the larger bulk material [2], besides showing an increase in surface area to volume ratio. In addition to their small size and large surface area, which becomes a very important feature in the nanoscale regime, constant developments over the past decade has made possible to manufacture them in a variety of different shapes, surface chemistry and core composition [3,4,5,6], controlling the design of nanoparticles (NPs) for drug delivery [7], imaging [8] and diagnostics [9] advancing in fields including cancer and immunotherapy, altogether aiming to help finding better strategies for diagnostics and treatment of several pathologies such as cancer, autoimmune diseases and so forth.

One important aspect in biomedical science is the detection of specific biological agents (such as tumour-associated antigens and other biomolecular markers) [10,11] in biological fluids for early-stage screening of diseases such as cancers as they can be asymptomatic until advanced stages, where the prognosis and survival rate are poor. In addition, much effort has been focused on the research involving the use of antibodies [12], which has made possible the production and purification of specific antibodies (such as monoclonal antibodies (mAbs)) against the desired antigen, opening a wide range of potential applications in many areas of research and health science, including the field of clinical diagnostics [13,14].

Thus, for this purpose immunosensors applying nanomaterials provide great advantages for clinical diagnostic and many other biomedical applications compared to conventional immunoassays (i.e., ELISA, Western Blotting, mass spectrometry-based proteomics, etc.), as they are based on a high specificity of the molecular recognition of antigens by antibodies, forming a stable immune complex and enhanced sensitivity.

### 1.1. Immune Complex: Antibody-Antigen Interactions

The immune system is one of the most complex and highly regulated biological responses and plays a major role in the protection of an organism against external pathogenic entities (such as bacterial or viral infections) but also in the detection and removal of aberrations in self-molecules, cells and tissues [15,16]. Both cases, conditions that could lead to morbidity and even death. It relies on two branches known as innate and adaptive response that differ in the time frame and specificity. While the innate immune system is a quick and broad response, the adaptive immunity arises later in time as a highly targeted response towards a particular threat. Complex networks of cooperating cells and biomolecules will result in the development of pathogen specificity by effector cells, such as cytotoxic T lymphocytes (CTLs) and biomolecules like antigen-specific antibodies [17].

**Figure 1:**
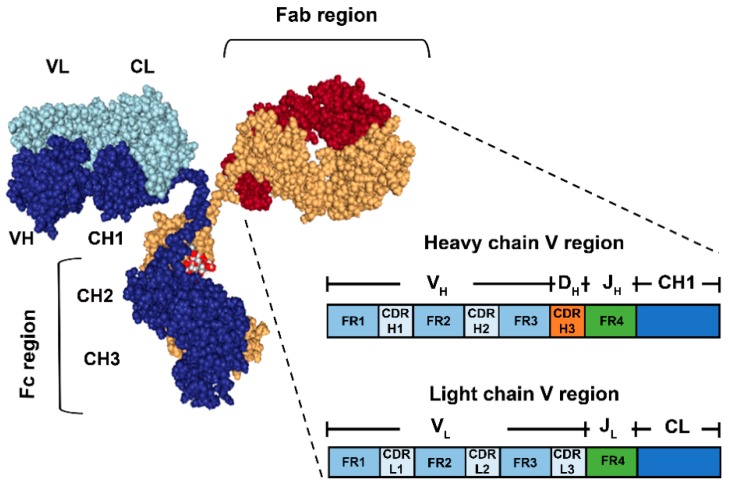
Schematic representation of the basic antibody structure (Immunoglobulin G) and detailed view of a Fab. The antibody is composed of two heavy chains (dark blue and yellow) and two light chains (light blue and red). The Fab regions contain the Variable fragments of the heavy and light chains (VH and VL respectively), where the antigen-binding site is located. The zoomed in view of the Fab shows the relevant regions contained in each of the H and L chains, namely Framework 1,2, 3 and 4 (FR1,2,3,4) and Complementarity-determining Regions 1, 2 and 3 (CDRH/L1,2,3) (Not shown at scale).It is indicated as well, which of the Ig gene segments (V(D)J) codify for each of the regions after somatic recombination and rearrangement during B cell maturation. On the other hand, the Fc region contains the H chain constant segments 2 and 3 (CH2,3).

Antibodies are glycoproteins that belong to the superfamily of immunoglobulins (Igs). The typical structure of an antibody is that of a Y-shaped molecule, comprised by two identical pairs of polypeptide chains known as heavy and light chain (Figure 1) linked by disulphide bonds. Typically, the antibody structure is divided into three regions: Fc (fragment crystallizable region) and two Fabs (fragment antigen binding region). The Fc region defines the Ig subclass, namely IgA, IgD, IgE, IgG and IgM. In the case of IgG, it is composed of the constant domains 2 and 3 (H2 and H3) of the heavy chain [13,18]. Each Fab contains the constant domains 1 (H1 of the heavy chain) and CL (constant domain of the light chain) and two variable domains (VL and VH, from light and heavy chain respectively) containing the antibody recognition and binding sites to a specific region of an antigen, known as epitope. It is believed that most of the specificity of an antibody for a particular epitope is contained in specific hyper-variable fragments of the V regions known as Complementarity-Determining Regions (CDRs), while the Framework Regions (FRs) flanking them are more conserved in sequence variability and believed to have minor roles in recognition but acting more as scaffolds for CDRs [19,20]. The latter are organized in a way that the CRDs 1, 2 and 3 of both heavy and light chain of both Fabs are in close proximity, allowing for a favourable topology for the accommodation of the epitope, which along to a range of non-covalent interaction will determine the specificity. The same way that the Fab has the function of recognizing and binding to the cognate epitope, the Fc region drives the other main effector function of the antibody molecule, recruitment and activation of cells and biomolecules in antibody-dependent responses, such as phagocytosis and antigen presentation, complement activation and degranulation [13,18].

The capability of producing almost infinite number of different antibodies to the plethora of individual pathogens, lays on the shuffling of gene segments contained in the Ig V(D)J (from Variable, Diversity and Joining) germline regions, in a process known as somatic or V(D)J recombination [21,22]. By the latter, the specific gene segments selected are recombined together to form the mature transcript that will be translated to the mature membrane-bound B cell receptor (BCR). B cells will undergo a process of clonal expansion and further modification of their BCR by introducing mutations in a process called Somatic Hypermutation (SHM). The resulting B cell clones which BCR shows high specificity for a particular antigen will maturate to Ig-secreting plasma cells. In this way, during an antibody response, multiple B cell clones are selected and will produce waves of antibodies. This process is usually known as a polyclonal response and it is characterized by antibodies with a wide range of specificities for different epitopes of one same antigen.

**Figure 2:**
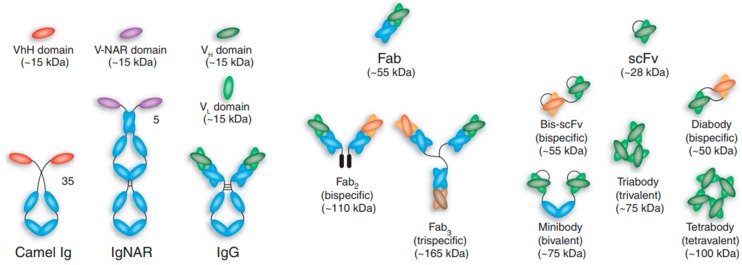
Schematic representation of different antibody formats, showing intact ‘classic’ IgG molecules alongside camelid VhH-Ig and shark Ig-NAR immunoglobulins. Camelid VhH-Ig and shark Ig-NARs are unusual immunoglobulin-like structures comprising a homodimeric pair of two chains of V-like and C-like domains (neither has a light chain), in which the displayed V domains bind target independently. Shark Ig NARs comprise a homodimer of one variable domain (V-NAR) and five C-like constant domains (C-NAR). A variety of antibody fragments are depicted, including Fab, scFv, single-domain VH, VhH and V-NAR and multimeric formats, such as minibodies, bis-scFv, diabodies, triabodies, tetrabodies and chemically conjugated Fab’ multimers (sizes given in kilodaltons are approximate). Reprinted with permission from Nature Biotechnology, 2005. 23: p. 1126. [25].

Of specific interest are those known as monoclonal antibodies (mAbs), which are produced by specific B cell clones and display high affinities for particular epitopes [23,24]. In the past few decades, the research involving antibodies has focused great attention due to the aforementioned capacities to specifically detect biomolecules, however, a key milestone in the development of antibody-based research and applications was the introduction of the myeloma method for the continued production of mAbs against the desired epitope. This opened a range of potential applications of such antibodies in many areas of research and medicine. Nowadays, immunoassays applying monoclonal antibodies are constantly used procedures in research and diagnostics. For instance, development of sandwich assays or their use for flow cytometry profiling are two of the main uses of monoclonal antibodies. And there are already examples of antibodies as therapeutic agents in immune therapy. Furthermore, advances in antibody engineering and phage display are constantly opening new opportunities, facilitating the production of a wide variety of antibodies and antibody-derived structures and fragments (Figure 2) with improved affinities by modifications of their CDRs [25]. Multiple formats of antibody are designed preserving their basic function of specifically detect the target, while substituting or removing specific parts that might not be necessary for the desired application, or even present deleterious effects towards the final application. For instance, several configurations lacking the Fc region, comprising only the antigen binding region of the antibody, have been produced in the search for smaller fragments with improved pharmacokinetics and tissue penetration (single chain antibody fragments, scAb) fulfilling and matching the requirements for an increasing number of applications and targets, for example, by increasing the sensitivity and reducing the cross-reactivity.

### 1.2. Nanoparticles for Biomarker Immunosensing

Many health disorders and diseases, including cancer, are typically asymptomatic and difficult to detect and diagnose until late stages when the effectiveness of treatments and survival rates are usually reduced [26,27]. Current diagnostic methodologies include different imaging tests (computed tomography, magnetic resonance imaging, X-ray imaging, etc.) that allow the detection of anatomical changes in healthy tissues associated to disease, or more invasive techniques such as direct tissue biopsy [26,28]. Despite the recent and continuous technological advances, the effectiveness of such methodologies for early diagnosis is still poor.

Most diseases present multiple development stages in which the alterations of normal cells and tissues have associated, similarly to healthy tissues, the production and secretion of specific biomolecules of different nature, which are nowadays referred to as biomarkers [28,29,30]. Such biomolecular patterns are prone to be profiled at different biological states to become informative and help decision making in terms of diagnosis and treatment. While the definition of biomarker was initially focused on proteins, nucleic acids and sugars, a deeper understanding of certain diseases has extended the concept to any measurable structure, including vesicles (exosomes) as they are known to carry a range of potential biomarkers. Some of the current examples of approved biomarkers for diagnosis are prostate-specific antigen (PSA) in prostate cancer or human epidermal growth factor receptor 2 (HER2) in breast cancer, in this latter case, being itself the target for therapeutic actions [31,32]. Thus, biomarker discovery and monitoring are of remarkable importance not only for diagnostics but to detect potential targets for alternative treatments. Biomarkers can be found in a range of different biological samples, such as urine, faeces or spinal fluid, however, the detection in the blood, by a simple blood test (liquid biopsy), is attracting a huge market due to ease of access and the low invasiveness [33,34,35,36]. Nowadays, the advances in high-throughput “omics” techniques, is key in the discovery of new candidates for human disease is one of the most growing fields in biomedical sciences and research [37].

**Figure 3:**
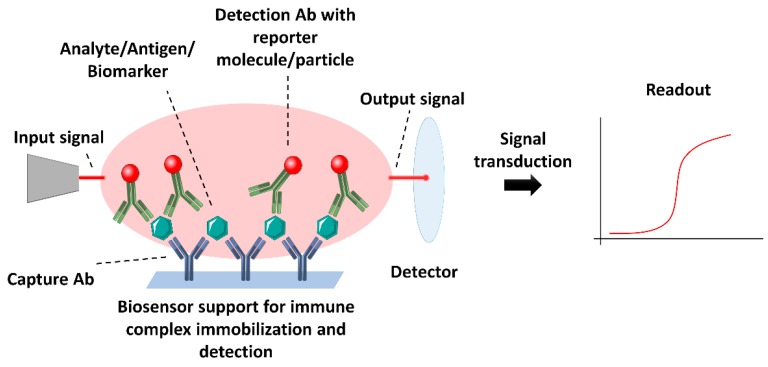
Schematic representation of the main components and mode of action of a immunosensor. In this case, a typical sandwich immunoassay-based biosensor is represented as an example of a biosensor setup. A device capable of immobilizing the molecule of interest for detection with a labelled specific antibody will allow the conversion of an input signal to a quantifiable output signal for transduction and generation of a final measurement.

Currently, specialized labs count on a variety of assays, techniques and instrumentation and well-trained staff for biomarker analysis from biological samples. However, besides the fact that some techniques still do not fulfil requirements, in some cases, the access to such techniques and expertise is limited, requiring a simpler and quicker approach. In this context, biosensors become a step forward in the development of cheap, fast and sensitive devices for diagnostics. Immunosensors allow the specific detection of the target biomarker from biological samples, transducing the presence of the specific analyte in the sample into a quantitatively measurable readout, such as chemi- or photo-luminic, colorimetric or electrochemical signal, depending on the nature and format of the biosensor [9,38,39,40,41,42,43,44]. In this way, antibodies and antibody-derived constructs play a key role in the development of more precise and specific, and with lower detection limits immunosensors. They base their application on the formation of the specific antigen-antibody complex for the detection and measurement of biomarkers and consequent production of a measurable signal by reporter molecules or mechanisms which are transduced to readable information (Figure 3). The most widely used immunosensors apply three different kinds of signal and transduction methods, following the formation of the complex antigen-antibody: optical, in which changes of the optical properties of the surrounding media are produced (e.g., colour, luminescence, changes in refractive index) [28,45,46]; electrochemical, based on electrical signals (current, voltage differences, resistance) [47,48,49] or piezoelectric, which rely on the changes in mass detected by piezoelectric devices [50]. Typically, most of the immunosensors are based on a sandwich type of assay, in which the antigen is captured by a capturing antibody and detected by a second antibody or detection antibody, which in most cases will carry the reporter molecule or agent.

The recent advances in nanotechnology, or the ability to manufacture NPs in precise ways, with very specific physical-chemical properties and highly tuneable surface modifications, has translated in the design of enhanced immunosensors by combining the specificity and versatility of antibodies and derivatives with the exploitation of intrinsic properties of NPs. This review focuses on highlighting some of the relevant applications of NP-based immunosensors and the key factors influencing their performance, from the different strategies for antibody immobilization for device construction, to the multiple formats based on the properties of the nanomaterials using their core composition as the distinctive parameter.

## 2. Nanomaterials for Immunosensing

Most biological samples extracted to be tested for specific biomarkers appear as highly complex media, with broad dynamic concentrations ranges of the different component biomolecules. This presents an issue from the point of view of background signal to noise ratios and specificity but also for the need of low detection and quantification ranges, as potential biomarkers may appear in low concentrations in such media. Therefore, lowering the detection limit is one of the key parameters when designing immunosensors, being one of the biggest advantages that NPs offer due to enhanced intrinsic properties.

NPs are the bridge between atoms and small molecules and the bulk material. Such nanosized objects present characteristic organization and behaviours of their atoms in their surface depending on the angle of curvature, which leads to a range of nanoscale effects. As a consequence, they show particular optical, mechanical and magnetic properties that are absent in their bulk counterparts and are highly dependent on their size and shape but also on the fabrication methods [2,3,4,5,6,7,8,9]. Such properties range from enhanced fluorescence, narrow emission peaks and high photostability (e.g., quantum dots), localized surface plasmon resonance bands and enhanced scattering properties (e.g., gold and silver NPs), catalytic activities (e.g., functionalized Graphene Oxide), superparamagnetism and so forth. Due to the advance in the synthetic procedures, it is nowadays possible to fabricate them in highly controlled and tuneable ways leading to highly reproducible size and shape distributions, thus, reproducible behaviours and properties. In addition to those arising as a result of the behaviour of their surface atoms, one intrinsic feature of NPs is their high surface area to volume ratio which increases as the core diameter of the NPs become smaller. This factor appears also crucial as it maximizes the efficiency of surface functionalization with specific groups/molecules/biomolecules in their surface per unit of volume of nanomaterial used in specific devices.

Due to the aforementioned characteristics of NPs, their use for immunoassay applications represent nowadays one of the most promising and extensively used detection approaches for the highly sensitive and selective immune detection of clinically relevant biomarkers. The biomolecular recognition elements, such as antibodies, are typically immobilized on the NP surfaces and their interaction with the analytes (i.e., antigens or molecular biomarkers) produce a physico-chemical response that modulates the signal. Therefore, precise control of the engineering of nanomaterials is also a key aspect to be considered in their application for immunosensing, as the main interactions occur on the interface between the NP and the biological environment [51]. When immobilizing antibodies, key aspects are preserving the activity of the molecule towards the cognate epitope and maximizing the accessibility of recognition sites, these aspects will be discussed further in the section. In addition, NPs for immunosensing must achieve specific conditions such as chemical stability in complex biological conditions, show very low (or none) toxicity and high signal transduction efficiency (i.e., the efficient signal capture of the antigen). Research on immunosensing using nanomaterials has centred on fluorescent quantum dots and plasmonic gold NPs and it has been also expanded to include carbon-based NPs, silicon NPs, among a constantly growing repertoire [52,53]. Their specific properties contribute to the development of label-free transduction techniques or contribute to signal amplification when used as labels [54].

### 2.1. Immobilization of Antibodies

Immobilization of the recognition elements to the NPs surface is one of the most important factors affecting NP-based immunosensors performance. Besides the preservation of the biological activity of the antibody, two other major parameters will determine the final performance of the immunosensor, that is, the orientation and the density of biorecognition molecules per unit of surface area of the NPs [55,56,57,58]. A correct orientation of the antibody will ensure that all, or most, recognition sites are presented in an accessible way for recognition of the epitope, on the other hand, a high density of grafted moieties will not only affect the efficiency but also will prevent non-specific interactions with other biomolecules from the samples and/or the device. These two factors will determine the limits of detection and overall performance of the immunosensor. Such parameters, including the preservation of the native form, will be directly influenced by the immobilization strategy followed. In this aspect, there is an immensely broad research, especially since the rise of nanotechnology for biomedical applications and in this review, we will briefly mention the most widely used along with some novel and highly efficient approaches.

The different ways of antibody immobilization on the NP surface are mainly dependent on the kind of interaction with the surface. In this way, antibodies can be immobilized by non-covalent or covalent interaction with the NP surface, by affinity or encapsulation. Depending on the different strategy for immobilization, the antibody molecules will present different levels of random or directed orientation of their antigen recognition sites. On the other hand, they also have different requirements in terms of time and costs of their preparation for the sensing devices.

Non-covalent immobilization includes adsorption by weak interactions such as electrostatic, hydrophobic or van der Waals forces. Such approach results the less costly and time consuming as it usually requires simple washing of NPs and incubation with the desired antibody, followed by removal of excess of antibody, however, this approach has multiple disadvantages, for instance, the known effects on the proteins stability by producing certain levels of denaturation and lower stability of the constructs, potential replacement by other molecules with the consequent loss of the antibody, besides being non-reusable devices due to the impossibility of regenerate by washing [59,60,61].

On the other hand, the most widely used approach in immobilization of antibodies to NPs and other surfaces is through covalent bonding. Such approach will result in longer lifetime devices, reusable and provide higher control over the orientation of the antibodies, allowing for higher detection capabilities. The presence of free amines (NH_2_) and thiol (–SH) groups in the antibody chain allow the use of different chemistries to form covalent bonds, being the most used ones carbodiimide chemistry, following the widely used EDC/NHS method by formation of an amide bond between the carboxylic group (–COOH) and the free amine groups (NH_2_) in the antibody and maleimide conjugation to thiol groups (–SH) [62,63,64]. Such approaches can be tuned to favour a specific orientation, however, their right orientation is not ensured. Another similar approach is by the known as click chemistry, which involves a variety of different groups and reactions that occur under specific conditions, being the most used one the Cu(I)-catalysed terminal alkyne-azide cycloaddition (CuAAC) [64,65].

Affinity approaches are based on the biorecognition of specific regions in the antibody by molecules on the surface of the NPs, it is the case of proteins A/G, that can recognize and anchor the antibody by the Fc region through non-covalent interactions [66]. Other approaches include the recognition of specific sugar residues in that same region. These approaches generate also oriented immobilization, on the other hand, they suffer from certain cross-reactivity and selectivity for certain molecules or Ig isotypes over others. Encapsulation of antibodies is one of the less used immobilization methods due to the highly reduced availability of recognition sites of the antibody.

Nowadays, antibody engineering allows for the precise design of the antibodies carrying specific groups or modifications using site-directed mutagenesis that will result in precise linkage with the NPs that can be adequately designed for such particular recombinant antibodies. This results in much higher control over the grafting orientation and density, however, producing such constructs appears significantly more expensive a time consuming than previous strategies [67].

### 2.2. Types of Nanomaterials for Immunosensing

#### 2.2.1. Carbon-Based Nanomaterials

**Figure 4:**
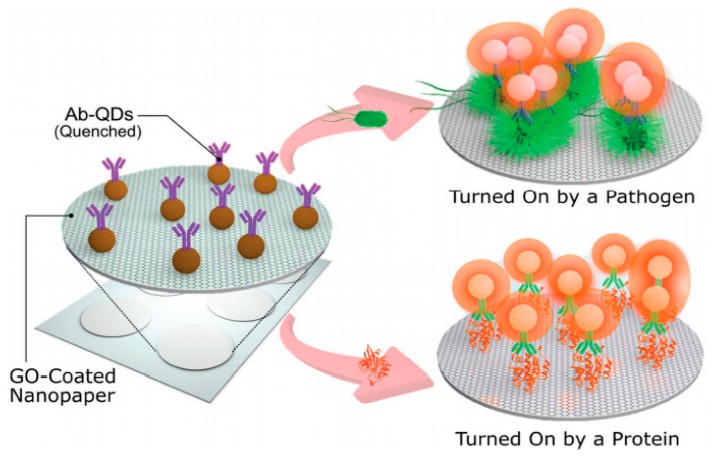
Operational concept of the immunosensing approach (schematic representation, not to scale). The hydrophilic, porous and photoluminescence-quenching character of GONAP allows for the adsorption and quenching of Ab-QDs, whereas photoluminescence recovery is triggered by the immunocomplex formation phenomenon, which involves a series of forces and interactions detaching the antigen-Ab-QD complex. Nevertheless, the antigen is then attached onto GONAP surface working as spacer between GONAP and Ab-QDs and hindering highly efficient nonradiative energy transfer. The immunosensing platform can be turned “On” by either big-sized analytes (pathogens) or small-sized analytes (proteins). Reprinted with permission from Advance Functional Materials, 2017. 27(38): p. 1702741. [69].

Graphene Oxide (GO) is obtained usually by exfoliation of graphite to generate two dimensional, single or few layers NPs in the form of graphene flakes formed by repetitive hexagonal structures of carbon atoms. The atomic structure of each carbon atom, *sp*^2^-hybrized with a perpendicular unhybridized Π-bond [68], gives to graphene nanostructures semiconductor properties which makes them highly photoluminescence (graphene quantum dots) and strong fluorescence quenchers by fluorescence resonance electron transfer (FRET) processes [69] making them suitable for bioassays relying on fluorescence signals. On the other hand, also present catalytic activity and high electric conductivity which results in the use for development of electrochemical devices. Generally obtained by oxidation of graphite, in the recent years has shown a great potential on bio-sensing platforms. Cheeveewattanagul et al. reported an immunosensing platform based on electron transfer and quenching of metallic quantum dots photoluminescence. By using a single antibody based on graphene oxide-coated nanopaper (GONAP), which facilitates the adsorption and quenching of photoluminescent quantum dots (QDs) nanocrystals conjugated with antibodies (ab) [69]. Upon immune complex formations (i.e., antigen-ab-QDs) the complexes go through a desorption process from GONAP surface and the photoluminescent of the QDs is then recovered, leading to a spacer (>20 nm) between ab-QDs and GONAP avoiding efficiently the transfer of non-radiative energy. Therefore, the QDs fluorescence recovery is proportional to the concentration of the analyte (Figure 4). Cheeveewattanagul et al. employed a model with human-IgG ab and Escherichia coli bacteria as analyte. In addition, series of immunoassays in higher complexity of biological environments were performed such as human serum, meat and water from the river to show the potential efficiency of the aforementioned approach. The application in complex biological environments of this type of immunoassays opens innovative capabilities in the analysis of food, environmental and biological samples. The work by Hwang et al. has also shown the quenching capabilities for the development of a cell surface immunosensor based on GO immobilized on the surface of living islet cells [70]. Fluorescein isothiocyanate (FITC) is chemically conjugated to GO via a linker peptide (Granzyme B-specific). The conjugated FITC is quenched by GO without any immune reaction, whereas it is dissociated from GO through proteolysis of the linker peptide when Granzyme B is secreted by immune cells such as natural killers (NK) cells and cytotoxic lymphocytes T. Therefore, the dissociated FITC recovers the fluorescence signal, demonstrating that there is an immune reaction, where Granzyme B is released by the cytotoxic lymphocytes T and NK cells. Further research in the field of cell surface immunosensors could help to monitor immune responses after transplantations and so forth.

GO nanomaterials can also be modified to show high catalytic properties that can be exploited in label free electrochemical devices. The work from Rauf et al. serves as example of immunoassay for cancer biomarker detection in complex media such as human serum. The modification of the GO surface with carboxyl groups (–COOH) provides intrinsic peroxidase activity reducing methylene blue (MB) and generating differences in current that can be measured by differential pulse voltammetry. In this case, antibodies conjugated to the GO detect Mucin 1 protein (MUC1), which is a membrane-associated glycoprotein and a well-known tumour biomarker found in a variety of malignant tumours [71]. Another example of electrochemical immunosensor based on graphene oxide is the presented by Singh et al., in which influenza virus H1N1 is detected in a microfluidic channel by recognition from specific antibodies immobilized in the surface of the nanomaterial [72]. The binding of the viral particles produces differences in the conductivity that can be translated into concentrations. The detection of tumour markers in biological fluids is crucial for early-stage screening of diseases such as cancers as they are usually asymptomatic until advanced stages. This type of immunosensors provides multiple applications in the field of clinical diagnostic and can be designed with several types of antibodies for various protein biomarkers.

#### 2.2.2. Metallic Nanomaterials

Gold Nanoparticles (AuNP) can be synthesized within a wide range of sizes and shapes with a multitude of surface coatings [43,47,73,74]. Moreover, they present characteristic localized surface plasmon resonance (LSPR) bands. Such surface plasmon resonance peaks are characteristics of metallic nanomaterials arising from the interaction of an electromagnetic field with the surface of the NPs producing a collective oscillation of the electrons, in the case of nanoscale objects highly accumulated and enhanced, leading to very efficient absorption and scattering of light across a broad spectrum ranging from the visible to the near-IR [75]. Such LSPR bands are characteristic for the specific sizes and shapes and can be tuned for specific applications but they are also modified by the properties of the surrounding media and modifications of the surface for example by binding events. In addition, size dispersion and morphology of AuNPs are easy to control and improve surface properties [41,43]. Altogether, make them excellent candidates for the development of novel immunosensors. In that sense, LSPR bands are highly sensitive to modification of the NP surface and surrounding media and can be directly used as immunosensing readouts. For instance, gold nanostars are formed by a central core from which a number of tips extend. Typically, they show multiple LSPR bands corresponding to the tips and core-tip interactions. Thus, streptavidin molecules can be detected upon binding to individual, biotin-modified gold nanostars by spectral shifts in the LSPR [75]. In a similar way Haddada et al. show a device for immune detection of staphylococcal enterotoxin A (SEA) based on plasmonic shifts of functionalized AuNPs upon binding to the target [76]. A similar format of immunoassay via aggregation or disaggregation is reported by Gupta et al. [77], where one population of AuNPs is coated with a protein-kinase substrate peptide and the other population of AuNPs is coated with the complementary anti-phosphotyrosine antibodies. The simultaneous addition of enzyme (Kinase) and ATP to the two populations of AuNPs, results in an enzymatic phosphorylation reaction of the AuNP-immobilized peptide substrate and cross-linking between NPs via specific recognition by the antibody-functionalized particles. As a result, changes in absorbance intensity at the plasmon resonance peak at 529 nm are observed. Moreover, aggregation processes are not observed in the absence of enzyme or ATP, which indicates that this reaction is a specific enzyme-driven response. Thus, the high selectivity and specificity of the immunoassays are important to minimize the risk of false positives.

Another approach for immunoassays is the use of surface-enhanced Raman scattering (SERS) spectroscopy by metallic nanoparticles since the discovery that the Raman scattering was enhanced in the proximity of nanomaterials [78]. A recent study showed a SERS-based immunosensor by employing spherical or nanorods AuNPs decorated with antibody fragments, non-fluorescent Raman-active dyes and passivating proteins [79]. In this case, the NPs serve as SERS enhancers due to plasmonic coupling upon formation of the antigen-antibody conjugate. This approach has been used in a multiplexed quantification of three different cytokines: interferon gamma (INFγ), interleukin-2 (IL-2) and tumour necrosis factor alpha (TNFα). Cytokines are peptides secreted by leukocytes and other cells that modulate physiological responses, including hematopoietic, inflammatory and immunity responses [80]. Thus, a NP mixture containing spherical and nanorods AuNPs for each cytokine (INFγ, IL-2, or TNFα) incubated with cell culture media harvested from CD4^+^ T cells, is measured by SERS spectroscopy. This approach allows a multiplex detection of relevant biomarkers such as cytokines, helping also in early detection of disease biomarkers and the development of tests for clinical diagnostics.

Plasmonic metallic nanoparticles, such as AuNPs, can be also applied for immunosensing based on their capabilities to enhance the fluorescence efficiency of fluorophores by non-radiative energy transfer processes [81], for instance, such processes have been studied by Yu et al. in the formation of immune complexes on a gold surface, enhancing the fluorescence signal of the labelled detection antibody. On the contrary, they can also be used as quenchers by FRET processes. Bu and co-workers [82] designed an immunosensor based on such phenomenon for determination of an organic pollutant by using AuNPs as quenchers of carbon dots (CDs) in a competitive immunoassay. Briefly, CDs functionalized with the pollutant, 4,4′-dibrominated biphenyl (PBB15), will be associated with AuNPs functionalized with specific anti-PBB15 antibodies, therefore quenching the fluorescence of the CDs. Upon addition of small amounts of the molecule of pollutant, this will compete for the antibody binding site and dissociate the CDs-AuNPs complexes, resulting in recovery of the fluorescence signal. Such effects of fluorescence enhancement and quenching are highly dependent on the distance between the plasmonic NP and the fluorophore and can be tuned for the specific applications [83].

Electrochemical immunosensors are one of the most widely used approaches in the design of highly sensitive and efficient detection devices due to the enhanced electrical signal amplification that the AuNPs provide due to their electron transfer capabilities to the electrode from the redox reactions occurring in the device [84]. In a recent publication, Alarfaj et al. exploited such conductivity properties of gold nanoparticles to enhance the detection of calcitonin (CTN), in combination with GO flakes [84]. Also, the AuNPs increase the surface area of the electrode, leading to increased amounts of capturing antibody immobilized. In a similar manner, both the electric conductivity properties and increased surface area available for immobilization that the AuNPs provide, Liu and colleagues developed a sensing device for atrazine, a known pesticide, showing low detection limits, reproducibility and recovery rates [85]. Such nanostructured electrodes are nowadays gaining huge attention due to their enhanced detection capacities. One recent example describing a capacitive immunosensor based on AuNPs is provided by Zeinabad et al. based on the immobilization of hepatitis B surface antigen by capturing antibodies functionalized in the surface of interdigitated electrodes and detection by AuNPs with immobilized capturing antibody [86].

Nanomaterials such as AuNPs and other plasmonic NPs can be used in lateral-flow and other colorimetric assays based on immobilization or aggregation of AuNPs for in vitro diagnostic immunoassays such as the pregnancy test based on the urine, to detect protein markers as humanchorionic gonadotropin (hCG) [41]. This protein introduced into a membrane interacts with AuNPs coated with anti-hCG antibody. The complex becomes attached to the surface of the membrane due to antigen-antibody interaction.

Silver Nanoparticles (AgNPs) are also metallic plasmonic nanoparticles and similarly to the AuNPs, they present specific LSPR bands which are depend on their size, shape and surrounded medium, making them also excellent NPs choice for the detection of biomarkers by immunoassays [87]. Many examples of NP-based immunosensors have been reported that rely on the plasmonic properties of AgNPs to detect specific biomarkers in biological samples [88] and are one of the most commonly used engineered NP due to their high capacity as antimicrobial agents to prevent infections [89]. However, even though the plasmonic and scattering properties are slightly higher in silver nanoparticles, they are usually substituted by gold nanoparticles due to their higher stability [90]. As a relevant example, triangular AgNPs conjugated with an anti-p53 antibody have been used to detect p53 serum protein in serum samples from head and neck squamous cell carcinoma (HNSCC) patients and in healthy control samples and detecting specific LSPR peak shifts [91]. The results suggest that the LSPR of this nanoimmunosensor assay based on triangular AgNPs is able to detect the p53 protein in the serum of HNSCC patient’s and illustrate the difference of serum p53 of the HNSCC patients and healthy control. The detection of p53 serum protein may play an important role in the diagnosis of a tumour, as it has been reported that levels of p53 serum protein were significantly increased in several carcinomas compared with normal controls [92].

Silver nanoparticles have also been used for the development of multiple electrochemical immunosensors. One example is based on their catalytic activity, in combination with cuprous oxide nanowires, for the reduction of hydrogen peroxide upon binding to an immobilized target protein (biomarker) through specific antibodies grafted in the surface of the nanomaterial [93]. In a similar manner to previous nanoparticles, AgNPs have also been used to maximize the immobilization of capture or detection molecules to enhance the performance of other electrochemical immunosensors [94].

**Figure 5:**
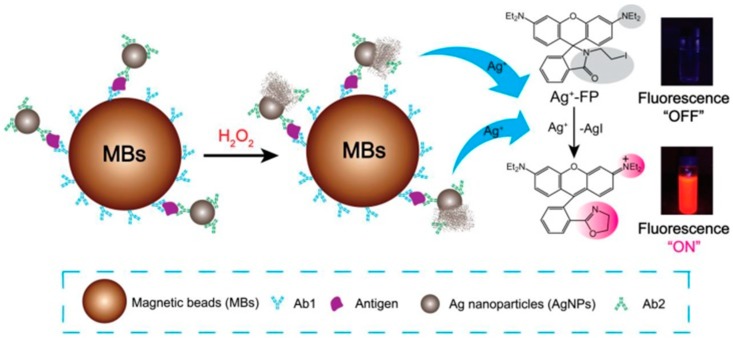
Schematic illustration of Ag^+^ triggered fluorescence detection for protein biomarkers. Reprinted with permission from Theranostics, 2017. 7(4) p. 876–883. [95].

In a different format, AgNPs-labelled antibodies can also be combined with separation techniques using magnetic beads to determine biomarkers based on a sensitive fluorescence detection system [95], where the fluorescence detection is improved using functionalized AgNPs. Each silver AgNP can be dissolved in the presence of hydrogen peroxide (H_2_O_2_) and produce millions of silver ions (Ag^+^), which turn “on” the fluorescence of the Ag^+^ fluorescence probe (Ag^+^-FP), increasing the fluorescence sensitivity of the designed immunoassay (Figure 5). Magnetic beads (MBs) are also applied in this system due to their remarkable properties, such as high specificity, facile separation and purification. Therefore, to detect a certain type of biomarker, after the analyte (i.e., antigen) is bound to Ab1-MBs, the Ab2-AgNPs complexes are added into the mixture and then the Ab2-AgNPs are captured on the surface of MBs via sandwich-type immuno-binding. The obtained immune complexes are collected via magnetic separation and the analyte-linked Ab2-AgNPs is dissolved in the mixed solution of Ag^+^-FP containing H_2_O_2_ to amplify the fluorescence signal. Such Ag^+^-triggered fluorescence detection system provides a promising platform for the detection of biomarkers that can be applied for clinical diagnosis.

**Figure 6:**
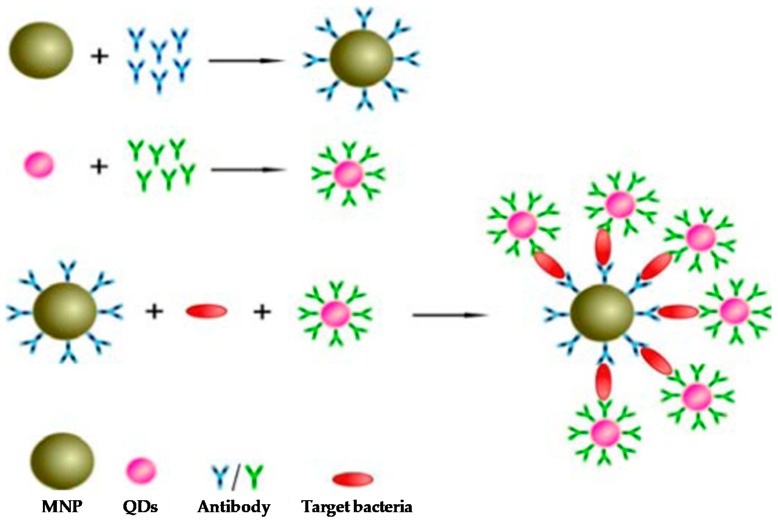
Schematic diagram of detection principle based on a sandwich assay using magnetic nanoparticles and quantum dots for Salmonella detection. Reprinted with permission from Int. J. Mol. Sci., 2013, 14(4), 8603. [99].

Quantum Dots (QDs) present numerous advantages over current fluorophores, such as organic dyes, fluorescent proteins and lanthanide chelates. Quantum confinement effects are observed, providing unique optical and electronic properties to QDs. Such effects produce the energy levels to be completely discrete and, upon excitation of electrons by incident light, photons are emitted at a specific wavelength when excited electrons return to ground states [96]. While conventional dyes have narrow excitation spectra, which requires excitation by light of a specific wavelength, QDs have broad absorption spectra, giving rise to excitation by a wide range of wavelengths. This property can be exploited to simultaneously excite multiple QDs of different colours by using a single wavelength. In addition, QD synthesis can be tailored to desired requirements, with specific core, shell and coating. Therefore, the high brightness and photo-stability and the narrow size-tuneable emission due to quantum confinement effects, make them highly suitable for sensitive multiplexed detection of several biomarkers with high selectivity, as the emission bands of QDs present less spectral overlapping compared to conventional fluorescent dyes [97,98]. Accordingly, QDs conjugated with antibodies (Abs) have often been employed for biological labelling to evaluate relevant biomarkers. For instance, QDs-Ab have been used to detect Salmonella, a common pathogen that causes food contamination and represents a significant threat to public health, with high sensitivity [99]. Two monoclonal antibodies are used to prepare Ab-coated magnetic NPs (i.e., immune-beads) and conjugated QDs-Ab. Thus, a sandwich structure is formed if Salmonella is mixed together with immune-beads and QDs-Ab, where the fluorescent signal from QDs is related to the amount of Salmonella (Figure 6). This type of immunoassay offers a great potential for the surveillance of Salmonella among another pathogen’s contamination. Moreover, several studies have also focused on the development of novel methodologies to map out the availability of recognition fragments of several proteins adsorbed on the surface of the NPs when they are in contact with biological fluids (a.k.a. Biomolecular-corona) using binding reporters, where Ab bound to QDs have been utilized for the detection of specific protein epitopes (i.e., specific molecular region of the protein -antigen- to which an Ab attaches itself) presented outwards on the surface of the NPs [100,101]. The signal from these highly fluorescent reporters can also be measured by Flow Cytometry [100]. Collectively, the application of these immunoassays to map out the surface of NPs highlights the biological identity of NPs in biological media (such as human plasma) and may hypothesize cellular receptor engagements and therefore the potential NP biological impact in cell systems [102].

Another important biological application of QDs involves Fluorescence Resonance Energy Transfer (FRET), where there is a transfer of fluorescence energy from a donor particle to an acceptor particle when the distance between donor and acceptor particles is smaller than Förster Radius [103]. For instance, Wang et al. have conjugated bovine serum albumin (BSA) to red-emitting CdTe NPs, while the corresponding anti-BSA antibody (IgG) is attached to green-emitting NPs [104]. Then, the formation of BSA-IgG immune complex results in FRET between the two different NPs, where the luminescence of green-emitting NPs has been quenched whereas the emission of the red-emitting NPs has been enhanced. This approach provides a detection as low as 10^−8^ M BSA with 5 × 10^−9^ M NP-BSA and NP-IgG. Moreover, the described method does not require the multiple binding and washing steps, unlike ELISA assay.

#### 2.2.3. Silicon Nanomaterials

**Figure 7:**
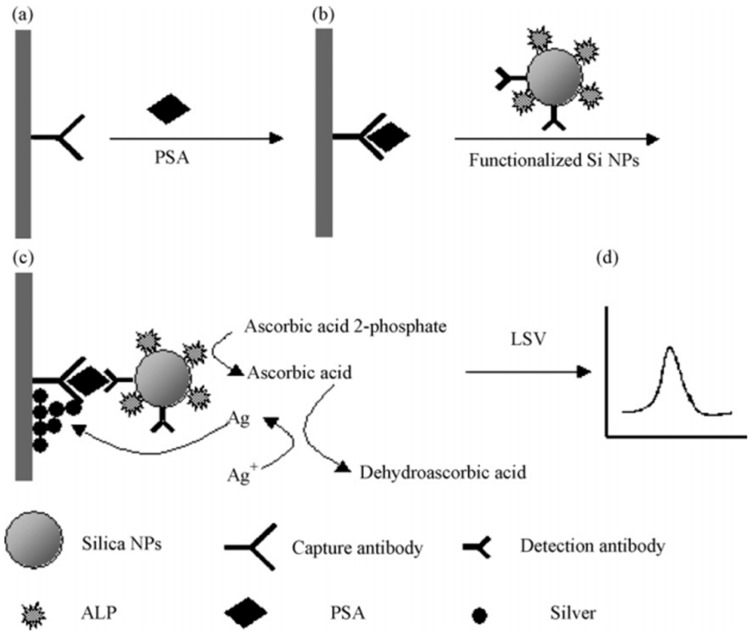
Schematic outline of the electrochemical immunosensor. (**a**) Immobilization of PSA capture antibody on the Au electrode; (**b**) capture of the analyte PSA in sample solution; (**c**) association with the functionalized silica NPs, reduction of silver ion by ascorbic acid and the deposition of metal silver on the electrode surface; (**d**) linear sweep voltammetry was used for the electrochemical detection of metal silver deposited on the electrode. Reprinted with permission from Talanta, 2008. 76(4): pp. 785–790. [107].

Silica Nanoparticles (SiO_2_ NPs) are considered an ideal biological probe due to the low toxicity of SiO_2_ NPs [105]. Developing sensitive and reliable sensing methods for the detection of clinical biomarkers in complex biological media has a potential in early detection of cancer, helping to the design of individual therapies [106]. Recently, mesoporous SiO_2_ NPs have gain interest in the biomedical field due to their unique properties, such as large surface area, high pore volume and controlled pore structure, allowing the attachment of several functional groups, including antibodies. For instance, Chen et al. reported an ultrasensitive chemiluminescence immunosensor for the detection of carcinoembryonic antigen (CEA) using HRP-functionalized mesoporous SiO_2_ NPs, where an amino-group functionalized mesoporous SiO_2_ NPs is used to simultaneously immobilize HRP and anti-CEA antibody on the surface of mesoporous SiO_2_ NPs [46]. Resulting in an enhanced sensitivity due to the signal amplification by a large amount of HRP introduced into the mesoporous SiO_2_ NPs, providing potential applications in clinical diagnosis. Qu et al. developed an electrochemical immunosensor based on functionalized SiO_2_ NPs for the detection of prostate-specific antigen (PSA) in human serum [107]. In this immunoassay, SiO_2_ NPs are co-functionalized with alkaline phosphatase (ALP) and the detection antibody; in addition, there is biocatalytic deposition of silver for an amplified electrochemical immunoassay of PSA in human serum (Figure 7). Thus, upon formation of sandwich immune complex of capture antibody, antigen (PSA) and the aforementioned functionalized SiO_2_ NPs on the electrode surface, the ALP carried on the SiO_2_ NPs converts the ascorbic acid 2-phosphate (AA-P) into ascorbic acid, which reduces Ag(I) ions in the solution, leading to deposition of silver onto the electrode surface. Ultimately, the amount of silver deposited onto the electrode surface is determined by linear sweep voltammeter (LSV). Providing a detection limit of human PSA of 0.76 ng/mL. Electrochemical immunosensors such as the latter one, are becoming one of the major techniques employed in the detection of biomolecules in the recent years, mainly due to their high sensitivity, relatively low cost and the requirement of small sample volume [108]. Yang et al. designed an immunosensor label based on functionalized mesoporous SiO_2_ NPs, where the electron transfer mediator (thionine (TH)) is encapsulated into the pore of mesoporous SiO_2_ NPs and the enzyme HRP and the secondary antibody (anti-human IgG antibody) are covalently conjugated onto themesoporous SiO_2_ NPs [109]. As a result, the designed immunosensor based on mesoporous SIO_2_ NPs–TH–HRP–antibody as label displayed a linear response for the detection of human IgG within a wide range of IgG concentration (0.01–10 ng/mL), a low detection limit and good reproducibility.

**Figure 8:**
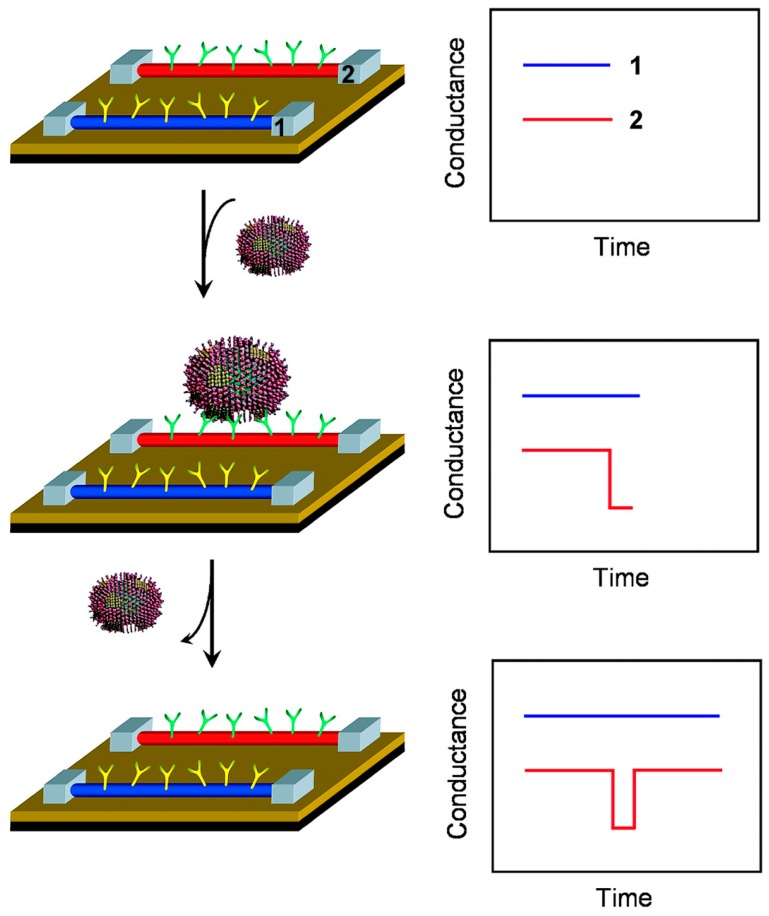
Nanowire-based detection of single viruses. (**Left**) Schematic shows two nanowire devices, 1 and 2, where the nanowires are modified with different antibody receptors. Specific binding of a single virus to the receptors on nanowire 2 produces a conductance change (**Right**) characteristic of the surface charge of the virus only in nanowire 2. When the virus unbinds from the surface the conductance returns to the baseline value. Reprinted with permission from PNAS, 2004. 101(39): pp. 14017–14022. [49].

Silicon Nanowires (SiNWs) have been also used for the development of high-performance immunosensors, taking advantage of their biocompatibility and high reproducible electrical and optical properties. Thus, SiNW immunosensors are generally based on field effect transistor (FET), made up of three electrodes [110]. The detection of viruses represents also an important aspect in health science, as they are one of the most important causes of human diseases. Thus, rapid, selective and sensitive detection is key to implement an effective response to viral infections. Patolsky et al. reported a direct, real-time detection of single virus particles with high selectivity based on SiNWs FET [49]. When a virus particle binds to the antibody on a nanowire device, the conductance of that device changes from the baseline value and when the virus unbinds, the conductance returns to the baseline value (Figure 8). Measurements made with nanowire arrays device modified with antibodies for influenza A showed discrete conductance changes, which are characteristic of binding and unbinding in the presence of influenza A virus but not in the presence of paramyxovirus or adenovirus. Moreover, further studies of nanowire devices modified with antibodies specific for either influenza A or adenovirus show that multiple viruses can be selectively detected in parallel. Therefore, these nanowire devices suggest a powerful tool for simultaneous detection of distinct viral threats at the single virus level.

## 3. Conclusions and Perspectives

As discussed previously in this review, nanomaterials-based immunosensors for in vitro and ex vivo applications can improve the readout of the assay in biological fluids compared with traditional methods, by lowering the detection limit and increasing physico-chemical signals. The vast variety of different materials, combinations and formats, provide flexibility for design of almost countless devices that can be carefully tuned for specific applications. And different modes of action are also continuously being developed. For instance, despite obvious advantages that highly fluorescence, plasmonic and scattering or highly reactive materials such as, QDs, AuNPs or GO provide in the immune detection of specific targets, different setups also take advantage from physico-chemical effects such as quenching or enhancing effects, that can provide better readouts. Moreover, another critical part of immunosensors is the biorecognition molecule, in this case antibodies or derivates, which should display high specificity and low cross-reactivity for the cognate antigen. In the past decades, the advances in research involving the use of antibodies is heading towards theproduction and purification of highly specific monoclonal antibodies and multiple different derivate antibody chains retaining the binding capacities but reducing unnecessary parts, combined with the fine control achieved over the design of NP-ab conjugates, along such advances in nanotechnology for manufacturing devices with highly specific antibodies, developments in the discovery of clinically relevant biomarkers from biological fluids leads to an increasing number of applications of devices in many areas of research and health science, that also includes the field of clinical diagnostics.

Current trends point out to the implementation of biosensing devices for continuous detection of biomarkers, in this sense nanotechnology allow the production of smarter NPs with improved and controlled properties and high reproducibility offers increased sensitivity for low abundance biomolecular species. On the other hand, allowing for development of faster and simpler devices that could improve their accessibility and applicability for non-expert users in many different areas.

However, there are still many issues and challenges in the field of nanomaterials-based biosensors for in vivo applications due mainly to incompatibility and toxicity issues related both to the materials used for fabrication but also to recognition of biological motifs in their surface [61] by combining the NP properties and current advances in nanomaterial science that, in addition to the development of immunology and proteome research will benefit the development of immunosensing technology, which can offer many opportunities to improve the effectiveness and the translation towards the clinical field, offering a wide range of advanced platforms application for clinical diagnosis and therapy, with extraordinary potential for health science and personalized medicine improving life, safety and security.
